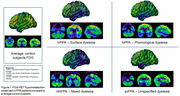# Navigating Dyslexia Diagnosis in Primary Progressive Aphasia within Transparent Languages: A Study in a Spanish‐Speaking Community

**DOI:** 10.1002/alz.090402

**Published:** 2025-01-03

**Authors:** Florentina Morello Garcia, Ismael Luis Calandri, Micaela A Hernández, Lucia Crivelli, Micaela Difalcis, Samanta Leiva, Aldo Ferreres, Ricardo Allegri

**Affiliations:** ^1^ Fleni, Buenos Aires, Buenos Aires Argentina; ^2^ University of Buenos Aires, Buenos Aires, Buenos Aires Argentina; ^3^ Alzheimer Center Amsterdam, Amsterdam UMC, Amsterdam Netherlands; ^4^ 'Eva Peron' Interzonal Acute Hospital, Buenos Aires, Buenos Aires Argentina

## Abstract

**Background:**

Surface dyslexia serves as a complementary feature in the classification of the semantic variant of Primary Progressive Aphasia (PPA), while reading deficits have also been reported in the other two PPA variants. In opaque languages, tasks involving regular and irregular words and non‐words are useful tools for dyslexia diagnosis. However, in transparent languages like Spanish, where most words are regular for reading, different approaches are needed. This study aims to: 1) present an alternative approach for detecting reading deficits in Spanish‐speaking PPA patients, and 2) identify brain FDG‐hypometabolism patterns in PPA subjects with dyslexia.

**Method:**

We assessed 17 PPA patients and 61 age‐sex‐education‐matched healthy subjects with tasks of word and non‐word reading, foreign word reading (FWRead), and visual lexical decision with pseudohomophones (VLDPsh). Employing a single‐case series methodology with a case‐control design, we conducted comparisons against controls and intra‐subject analyses. To assess hypometabolism, we performed 18F‐FDG PET scans on all subjects, and we analyzed the images using SPM. We adjusted a prediction model of 18F‐FDG signal in control subjects to determine the w‐score for each patient. A region was defined as abnormal if it fell below a w‐score of ‐1.5.

**Result:**

We detected dyslexia in 94% of all PPA subjects. Specific patterns were identified: 5 cases of surface dyslexia (characterized by impairments in the lexical reading mechanism), 2 of phonological dyslexia (impairments in the phonological mechanism), 3 of mixed dyslexia (impairments in both reading mechanisms), and 6 unspecified. The FDG‐PET results revealed left ventral occipitotemporal, left ventral inferior parietal, and left superior temporal cortex hypometabolism (see Figure 1).

**Conclusion:**

This research provides valuable insights into the presence and types of dyslexia shown by Spanish‐speaking PPA patients and emphasizes the importance of studying reading, as it has been observed to be affected in 94% of our sample. Our findings underscore the need to employ tasks such as FWRead and VLDPsh to assess lexical reading mechanisms in Spanish‐speaking contexts. Moreover, the identification of hypometabolism in brain regions contributes additional insights into the metabolic deficits observed in PPA patients and is consistent with reported data about regions linked to reading.